# Effect of individual- versus collective-based nutritional-lifestyle intervention on the atherogenic index of plasma in children with obesity: a randomized trial

**DOI:** 10.1186/s12986-020-00537-w

**Published:** 2021-01-13

**Authors:** Elvira Verduci, Giuseppe Banderali, Elisabetta Di Profio, Sara Vizzuso, Gianvincenzo Zuccotti, Giovanni Radaelli

**Affiliations:** 1 Department of Health Sciences, University of Milan, Via Antonio di Rudinì 8, 20142 Milan, Italy; 2 Department of Paediatrics, Vittore Buzzi Children’s Hospital, University of Milan, Milan, Italy; 3 Department of Paediatrics, San Paolo Hospital, University of Milan, Milan, Italy

**Keywords:** Atherogenic index of plasma, Children, Nutritional intervention, Obesity

## Abstract

**Background:**

The Atherogenic Index of Plasma is a predictive biomarker of atherosclerosis in adults but there is a lack of studies in paediatric population aimed at evaluating the longitudinal changes of the AIP and of the cardiometabolic blood profile related to nutritional interventions. The aim of this study was to compare the effect of individual- versus collective-based nutritional-lifestyle intervention on the Atherogenic Index of Plasma in schoolchildren with obesity.

**Methods:**

One-hundred sixty-four children aged 6–12 years with Body Mass Index z-score > 2 referred to the Paediatric Obesity Clinic, San Paolo Hospital, Milan, Italy, were consecutively enrolled and randomized to undergo to either an individual- (n = 82) or a collective- (n = 82) based intervention promoting a balanced normo-caloric diet and physical activity. In addition, the individual intervention included a tailored personalized nutritional advice and education based on the revised Coventry, Aberdeen, and London-Refined taxonomy. Both at baseline and after 12 months of intervention, dietary habits and anthropometric measures were assessed, a fasting blood sample were taken for biochemistry analysis.

**Results:**

The participation rate at 12 months was 93.3% (n = 153 patients), 76 children in the individual-intervention and 77 children in the collective intervention. At univariate analysis, mean longitudinal change in Atherogenic Index of Plasma was greater in the individual than collective intervention (− 0.12 *vs*. − 0.05), as well as change in triglyceride-glucose index (− 0.22 *vs.* − 0.08) and Body Mass Index z-score (− 0.59 *vs.* − 0.37). At multiple analysis, only change in Body Mass Index z-score remained independently associated with intervention (odds ratio 3.37).

**Conclusion:**

In children with obesity, an individual-based nutritional and lifestyle intervention, including techniques from the CALO-RE taxonomy, could have an additional beneficial effect over a collective-based intervention, although the actual size of the effect remains to be clarified.

*Trial Registration *Clinical Trials NCT03728621

## Introduction

Obesity rates have risen rapidly worldwide over the past few decades [1, 2] and obesity is currently one of the most serious global public health burdens [3]. Children with obesity may exhibit metabolic profile derangement [4] and an increased cardiovascular risk [5] lasting possibly even in adulthood [4]. Obesity-related atherogenic dyslipidaemia is an adverse risk factor for cardiovascular disease in children [6] that may be associated with structural and functional vascular changes [7], thus increasing the risk of early detrimental health outcomes [7] and later cardiovascular events [8]. The Atherogenic Index of Plasma (AIP) is a major indicator of the size of pre- and anti-atherogenic lipoprotein particles. It is today considered both a predictive biomarker of atherosclerosis risk in adults [9, 10] and a better indicator than others lipid variables in assessing cardiovascular risk [11, 12], particularly associated with obesity [13].

In both overweight and obese children, nutritional interventions are a crucial step recommended by the international guidelines to profitably manage obesity [4, 14] also because those could result in improvement in the status of obesity [15–17] and cardiometabolic blood profile [15, 18]. Despite the potential clinical and social relevance of nutritional interventions, there is paucity of studies in the paediatric population evaluating their effectiveness in outpatients [19, 20]. Moreover, although Hayes et al. [19] suggested that interventions including an individual approach instead of collective nutritional advice may result in better improvement of body weight, there is a lack of randomized trials investigating this hypothesis and any effect on metabolic blood profile.

The primary aim of this study was to evaluate the effect of an individual-based nutritional-lifestyle intervention, compared to a collective one, on the atherogenic plasma index in schoolchildren with obesity. Secondarily, the effect on Body Mass Index (BMI) was investigated.

## Material and methods

### Settings and participants

This was a longitudinal parallel randomized open trial with an allocation ratio 1:1. Eligibility criteria for participants were as follows. Inclusion criteria: BMI z-score > 2, based on the WHO references [21], age 6 – 12 years, gestational age 37–42 weeks, body weight at birth ≥ 2500 g and < 4000 g, single birth, white parents, family living in Milan or neighbourhood (≤ 30 km). Exclusion criteria: child having syndromic, organic and/or hormonal conditions besides obesity, child on a diet and/or medication that could affect body weight, child needing hospital admission. The data were consecutively collected among children whom the family paediatrician had first addressed for suspected obesity to the Paediatric Obesity Clinic, Department of Paediatrics, San Paolo Hospital, Milan, Italy. At the first ambulatorial general visit, the eligibility criteria were assessed, anamnestic data were recorded, and general routine instructions and recommendations were given to parents and children in accordance with the standard Good Clinical Practice guidelines. The parents were asked if they would be willing to make their child participate in a trial based on specific nutritional and lifestyle interventions, scheduled to start within six months. The parents or legal guardians received a detailed explanation about the aim and design of the study and were requested to sign a consent form. The trial was registered at clinicaltrials.gov (Trial number NCT03728621). Baseline trial data were collected one day before starting intervention, when eligibility criteria were reassessed, and 12 months later, and included anthropometric measurements, dietary habits, physical activity and biochemical evaluations.

### Interventions

The study protocol planned to administer the participants to either an individual- or a collective-based intervention promoting a normo-caloric diet and physical activity. Both in individual and collective intervention, general dietary recommendations were balanced and age- and sex-adjusted in accordance with the national guidelines for treatment of childhood obesity [22] and the Italian Society of Human Nutrition [23], that are Daily Energy Intake (En) 1372–2499 kcal (depending on age and sex); protein: 0.94–0.97 g/kg/die; carbohydrates: 45%-60% En, sugar < 15% En; fats: 20%-35% En (of which saturated fatty acids < 10% En, polyunsaturated fatty acids 5–10% En); fiber: 8.4 g/1000 kcal [22, 23]. Any child was further requested to engage in at least 60 min of moderate to vigorous daily physical activity (MVPA) [24]. The children and their parents were trained in the hospital during an educational two-hours session held by the same expert paediatrician assisted by a nutritionist. The training course was organized to be held in a class of 4 children (collective intervention) or individually (individual intervention), and instructed them and parents about regulation of energy expenditure, body composition, physical activity, consequences of sedentary lifestyle, principles of nutrition, food sources, glycaemic index and glucose metabolism. In the individual intervention, recommendations were refined and personalized on the child's preferences regarding food and lifestyle, and a series of supplementary behaviour modification techniques were recommended in accordance to the Coventry, Aberdeen and London-Refined (CALO-RE) taxonomy (items 1, 2, 5, 6, 8, 16, 21, and 26) [25]. Written guidelines were given to the parents, including general nutritional advice, food choice lists, recommended average servings for principal food categories, according to Italian Dietary Reference Values [23]. General nutritional advices included increasing fruit and vegetables (> 400 g/die, or five portions [26]), legume and fish intakes while decreasing meat consumption, avoiding sugar-sweetened beverages and limiting sweets and introducing more whole grain food, also according to the principles of the Mediterranean diet [27]. An illustrated brochure explaining potential benefits of daily physical activity and a diary for daily recording physical activity of the child in terms of type, frequency, duration and intensity were given to parents. The paediatrician invited parents to contact the hospital staff throughout the intervention period when needed or desired, in any case whenever any adverse event occurred. The educational session was repeated 6 months after starting the intervention. Compliance with nutritional intervention was estimated at 12 months by the rate of daily dietary energy intake within the recommended range, and compliance with physical activity by the rate of MPVA longer than 60 min.

### Anthropometry

The body weight and height of any child were measured using a mechanical column scale (SECA 711; SECA GmbH & KG, Hamburg, Germany) with an integrated measuring rod (SECA 220; SECA GmbH & KG). Body Mass Index (BMI) and related z-scores were calculated from the ratio of weight to height squared (kg/m^2^) using the WHO reference growth charts [21]. Puberty stage was determined.

### Dietary habits

Dietary habits of children were assessed by a validated Italian language food frequency questionnaire (FFQ) developed on the original Block FFQ [28], and then updated in 2008 on the basis of the full-length Block 2005 FFQ© (NutritionQuest, Berkeley, CA, USA) and the 2007 new national food composition tables [29]. Parents completed the FFQ during an interview of approximately 50 min, conducted by the same nutritionist. Each meal was analysed to find out which food was eaten and how often. Usual portion sizes were estimated using household measures and the weight (*e.g.*, pasta) or unit (e.g. fruit juice) of the purchase. A 24-h recall was additionally recorded at the end of the interview to standardize the usual serving size. Quantification and analysis of the energy intake and nutrient composition were performed with an ad hoc PC software developed by a consultant.

### Biochemistry

Fasting blood samples were taken at 8.00 ± 30 min a.m. and immediately analysed at the hospital laboratory of biochemistry for total cholesterol, high-density lipoprotein (HDL) cholesterol, low-density lipoprotein (LDL) cholesterol, triglycerides (TG), apolipoprotein A1 (ApoA1), apolipoprotein B (ApoB), glucose and insulin (cobas® 6000 analyzer series, c501 and e601 module, Roche Diagnostics GmbH, Hoffmann-La Roche ltd, Mannheim, Germany). The AIP was calculated as log10 of plasma triglycerides (mg/dL)/HDL-cholesterol (mg/dL) [9]. The homeostatic model assessment of insulin resistance (HOMA-IR) was calculated as the product of fasting glucose (mmol/L) and fasting insulin (U/mL) divided by 22.5 [30], and insulin resistance was defined as HOMA-IR > 3.16 [31]. The homeostatic model assessment of β-cell function (HOMA-β%) was evaluated as [20 * fasting insulin(U/mL)/fasting glucose (mmol/L)—3.5] [30]. The Quantitative Insulin Sensitivity Check (QUICK) index was calculated as 1/[log10 fasting plasma insulin (U/mL) + log10 glucose (mg/dL)] [32]. The triglyceride-glucose (TyG) index was calculated as ln[triglycerides (mg/dL) /glucose (mg/dL)/2] [33].

### Outcomes measures

The primary outcome measure was the change in the AIP after 12 months of starting intervention. The secondary outcome measures were changes in the TyG, HOMA-IR and BMI z-score. No change to trial outcomes was made after the trial started.

### Sample size

The sample size was calculated to detect a difference of 0.5 SD or more in the change of the AIP. Assuming a type I error level of 0.05 with a power of 0.80 and a 12 months drop-out rate of 15%, at least 76 children needed to be recruited for each group.

### Randomization

Children were randomly allocated to individual or collective intervention by a computer generated, blocked list. A block size of four was used, stratified according to age (± 6 mo.) and sex. The random allocation sequence was implemented sequentially in concealed electronic tables by an informatic blinded to the study. The tables were stored and masked on a single computer. Upon enrolment of a child, the recruiting paediatrician accessed the computer and then the electronic tables via a system-generated numeric code sent to its phone and valid only once for 15 s. By selecting the specific age and sex table, the first sequentially available still masked tile was revealed, and the paediatrician was made aware about the allocation group. The system automatically prevented the paediatrician from selecting any other still masked cells and reading the previously used tiles.

### Statistical analysis

Descriptive data are reported as mean, standard deviation (SD) or number of observations (percentage). The 95% confidence interval (CI) was reported as appropriate. Crude significance of within-group and inter-groups differences were tested by the Student’s t test or the Wilcoxon test or the Mann–Whitney U test, as appropriate. A multiple stepwise logistic regression model was fitted (enter at *p* < 0.15, remove at *p* > 0.20) to assess if an independent association may exist of change in AIP with the type of intervention. Covariates entered into the model were: age, sex, Tanner stage, baseline dietary energy intake and macronutrients, any examined blood lipid or glucose metabolism variable or index. Two dummy variables were also constructed and included that are compliance at 12 months with dietary energy intake (yes/no) and with MPVA (yes/no). Values of *p* < 0.05 were considered to indicate statistical significance (two-tailed test). The IBM SPSS Statistics for Windows, Version 25.0, (IBM Corp. Released 2017, Armonk, NY) was used for statistical analysis.

## Results

**Fig. 1:**
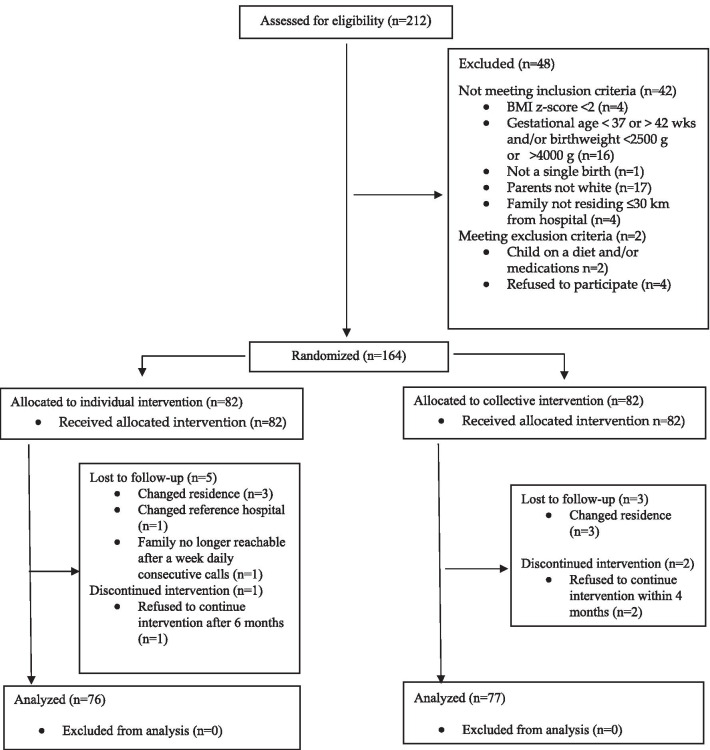
CONSORT flow diagram of the trial

Figure 1 shows the participant flow of the study. Participants were consecutively recruited, from January 01, 2016, to June 30, 2018, and follow-up ended on December 31, 2019. The participation rate at 12 months was 93.3% (n = 153). Preliminary analysis disclosed no difference in any baseline anthropometric, dietary or biochemistry variable between children who completed the trial and those did not (minimum *p* = 0.173). Participants at 12 months were included in all analysis, and the analysis was by original assigned groups, that is 76 and 77 in the individual or collective intervention group, respectively.

Table 1 shows the baseline characteristics for each intervention group. The daily energy intake was higher than the recommended in 23.7% *vs.* 27.3% of children in individual *vs.* collective intervention group, while protein, carbohydrates and fats (as % of energy intake) were outside the recommended values in 42.1% *vs.* 45.4%, 31.6% *vs*. 27.3% and 13.2% *vs.* 14.3%, respectively. No difference occurred between groups (minimum *p* = 0.559). After 12 months of intervention, energy, macronutrients and fiber intakes, as well MVPA recovered to the recommended values in both groups (minimum *p* < 0.001) and no difference was observed between groups (minimum *p* = 0.104). Compliance with the intervention was 90.8% and 88.3% in children who underwent individual or collective intervention respectively (*p* = 0.617), while compliance with MVPA was 85.5% and 83.1% (*p* = 0.821).

**Table 1:** Baseline anthropometric characteristics, daily dietary intake and MVPA

Variable	Individual intervention (n = 76)	Collective intervention (n = 77)	*p* value	Recommended^†^
Age (years)	10.0 (2.4)	9.9 (2.3)	0.775	
Sex (males)	37 (48.7%)	36 (46.7%)	0.753	
Dietary intake				
Energy				
kcal	2721.7 (970.6)	2590.2 (940.3)	0.3959	1372–2499 kcal/day
kJ	11,387.59 (4060.99)	1,083,740 (3934.21)		depending on age and sex
Protein				
g	104.8 (33.8)	103.3 (34.5)	0.7862	
% Energy	15.4 (3.1)	15.9 (3.4)	0.3435	< 15% Energy
Carbohydrates				
g	380.3 (150.4)	353.0 (153.07)	0.1495	
% Energy	55.7 (5.5)	54.6 (5.6)	0.1232	45–60% Energy
Fats				
g	97.6 (39.8)	95.6 (39.4)	0.7552	
%Energy	28.7 (4.3)	29.4 (4.7)	0.2237	20–35% Energy
Fiber				
g	15.12 (6.91)	14.78 (7.23)	0.7666	
g/1000 kcal	5.567 (1.94)	5.706 (2.01)	0.6618	8.4 g/1000 kcal
MVPA (min/day)	43.8 (31.3)	45.0 (32.4)	0.803	> 60 min

Values are mean (SD), median (min–max) or number of observation (%)

*MVPA* moderate to vigorous daily physical activity

^†^Recommendations in accordance with References [22–24]

Blood lipid and glucose metabolism profiles for each group at baseline and after 12 months of intervention are shown in Tables 2 and 3 respectively. No difference between groups was observed for any lipid variable except total and LDL cholesterol. Triglycerides decreased in both groups, but a reduction of the AIP was found in the individual group only (Table 2). No difference between groups was observed for any variable of the glucose metabolism but a significant reduction of the TyG index was found in the individual intervention group only (Table 3). No difference between groups was found for the rate of insulin resistance neither at baseline (48.7% *vs*. 37.7%, *p* = 0.169) nor at 12 months (39.5% *vs*. 32.5%, *p* = 0.347). Longitudinal change in rate was − 9.2% (95% CI − 17.5 to − 0.91%, *p* = 0.065, McNemar test) and − 5.2% (95% CI − 11.3 to 0.93%, *p* = 0.219, McNemar test) in individual and collective group respectively, and it did not differ between the groups (*p* = 0.336).

**Table 2:** Blood lipid profile at baseline and 12 months of intervention

Variable	Individual intervention (n = 76)	Collective intervention (n = 77)	*p-*value
Total cholesterol (mmol/L)			
Baseline	4.40 (4.20 to 4.60)	4.01 (3.80 to 4.21)	0.015
12 months	4.20 (4.00 to 4.40)	3.92 (3.73 to 4.11)	0.007
*p-*value^†^	0.001	0.108	
LDL cholesterol (mmol/L)			
Baseline	2.58 (2.42 to 2.74)	2.42 (2.21 to 2.62)	0.030
12 months	2.50 (2.32 to 2.68)	2.25 (2.05 to 2.45)	0.012
*p-*value	0.023	< 0.001	
HDL cholesterol (mmol/L)			
Baseline	1.29 (1.23 to 1.35)	1.24 (1.18 to 1.30)	0.161
12 months	1.32 (1.26 to 1.38)	1.25 (1.19 to 1.31)	0.142
*p-*value	0.117	0.413	
Triglycerides (mmol/L)			
Baseline	1.19 (1.07 to 1.31)	1.10 (0.97 to 1.23)	0.126
12 months	0.90 (0.82 to 0.98)	0.95 (0.86 to 1.04)	0.548
*p-*value	< 0.001	0.021	
Apo A1 (g/L)			
Baseline	1.33 (1.28 to 1.38)	1.37 (1.29 to 1.45)	0.663
12 months	1.33 (1.28 to 1.38)	1.35 (1.31 to 1.39)	0.310
*p-*value	0.978	0.193	
Apo B (g/L)			
Baseline	0.76 (0.71 to 0.81)	0.76 (0.70 to 0.82)	0.496
12 months	0.75 (0.70 to 0.80)	0.73 (0.68 to 0.78)	0.269
*p-*value	0.892	0.322	
AIP			
Baseline	0.29 (0.23 to 0.35)	0.27 (0.21 to 0.33)	0.463
12 months	0.17 (0.12 to 0 to 22)	0.22 (0.17 to 0.27)	0.230
*p-*value^†^	< 0.001	0.100	

Values are mean (95% CI)

*LDL* low to density to lipoprotein, *HDL* high to density to lipoprotein; *Apo A1* apolipoprotein A1, *Apo B* apolipoprotein B, *AIP* atherogenic index of plasma

**Table 3:** Glucose metabolism profile at baseline and 12 months of intervention

Variable	Individual intervention (n = 76)	Collective intervention (n = 77)	*p-*value
Glucose (mmol/L)			
Baseline	4.76 (4.69 to 4.83)	4.76 (4.64 to 8.88)	0.593
12 months	4.73 (4.66 to 4.79)	4.58 (4.52 to 4.66)	0.404
*p-*value	0.597	0.273	
Insulin (pmol/L)			
Baseline	122.93 (104.38 to 141.49)	120.98 (100.43 to 141.53)	0.347
12 months	99.87 (85.69 to 112.05)	100.63 (85.10 to 116.16)	0.615
*p-*value	0.003	0.009	
HOMA to IR			
Baseline	3.78 (3.09 to 4.26)	3.68 (2.98 to 4.37)	0.190
12 months	3.06 (2.63 to 3.49)	3.54 (2.87 to 4.21)	0.830
*p-*value	0.005	0.662	
HOMA to β%			
Baseline	291.92 (247.59 to 366.25)	335.49 (275.87 to 395.11)	0.334
12 months	233.32 (206.29 to 260.35)	251.98 (219.05 to 284.92)	0.773
*p-*value	0.010	< 0.001	
QUICK index			
Baseline	0.32 (0.31 to 0.33)	0.33 (0.32 to 0.34)	0.161
12 months	0.33 (0.32 to 0.34)	0.33 (0.32 to 0.34)	0.492
*p-*value	0.007	0.156	
TyG index			
Baseline	8.29 (8.18 to 8.39)	8.18 (8.07 to 2.89)	0.116
12 months	8.07 (7.98 to 8.16)	8.10 (8.01 to 8.19)	0.668
*p-*value	0.003	0.124	

Values are mean (95%CI)

*HOMA to IR* homeostatic model assessment of insulin resistance, *HOMA to β%* homeostatic model assessment of β to cell function, *QUICK* quantitative insulin sensitivity check, *TyG* triglyceride to glucose

The longitudinal change of lipid and glucose metabolic variables for each group is shown in Table 4. Mean absolute change was higher in individual compared to collective intervention for the AIP (difference − 0.07, 95% CI, − 0.14 to − 0.02), TyG index (difference − 0.14, 95% CI − 0.26 to − 0.02) and triglycerides (difference -0.14, mg/dL 95% CI − 0.28 to − 0.006).

**Table 4:** Longitudinal change (12 months—baseline) of blood glucose metabolism and lipid profile

Variable	Individual intervention (n = 76)	Collective intervention (n = 77)	*p*-value
*Lipid*			
Total cholesterol (mmol/L)	− 0.20 (− 0.35 to − 0.05)	− 0.09 (− 0.004 to 0.18)	0.056
LDL cholesterol (mmol/L)	− 0.08 (− 0.17 to − 0.001)	− 0.17 (− 0.27 to − 0.07)	0.359
HDL cholesterol (mmol/L)	0.03 (− 0.02 to 0.08)	0.01 (− 0.03 to 0.05)	0.537
Triglycerides (mmol/L)	− 0.29 (− 0.38 to − 0.20)	− 0.15 (− 0.24 to − 0.03)	0.036
Apo A1 (g/L)	− 0.002 (− 0.05 to 0.05)	− 0.02 (− 0.10 to 0.06)	0.512
Apo B (g/L)	− 0.01 (− 0.08 to 0.06)	− 0.03 (− 0.08 to 0.02)	0.460
AIP	− 0.12 (− 0.16 to − 0.08)	− 0.05 (− 0.10 to 0.005)	0.013
*Glucose metabolism*			
Glucose (mmol/L)	− 0.02 (− 0.11 to 0.07)	0.08 (− 0.002 to 0.16)	0.156
Insulin (pmol/L)	− 23.06 (− 38.39 to − 7.73)	− 20.35 (− 37.65 to − 3.06)	0.804
HOMA-IR	− 0.72 (− 1.21 to − 0.22)	− 0.21 (− 0.89 to 0.47)	0.095
HOMA-β%	− 58.60 (− 92.20 to − 25.00)	− 84.84 (− 142.74 to − 29.44)	0.390
QUICK index	0.01 (0.005 to 0.014)	0.001 (− 0.006 to 0.01)	0.447
TyG index	− 0.22 (− 0.30 to − 0.14)	− 0.08 (− 0.01 to 0.17)	0.005

Values are mean (95% CI)

*LDL* low-density-lipoprotein, *HDL* high-density-lipoprotein, *Apo A1* apolipoprotein A1, *Apo B* apolipoprotein B, *AIP* atherogenic index of plasma, *HOMA-IR* homeostatic model assessment of insulin resistance, *HOMA-β%* homeostatic model assessment of β-cell function, *QUICK* quantitative insulin sensitivity check, *TyG* triglyceride-glucose

Through the 12 months of intervention, mean BMI z-score decreased both in the individual and collective intervention group (*p* < 0001). The mean longitudinal change was higher in individual intervention group (*p* < 0.0001) (Table 5), with an absolute mean difference between groups of 0.22 (95% CI 0.09 to 0.35). BMI z-score decreased to below 2 in 7 (9.2%, binomial 95% CI 3.8 to 18.1%) and 6 (7.8%, binomial 95% CI 2.9 to 16.3%) of children in individual and collective intervention groups, respectively.

**Table 5:** Body mass index z-score at baseline and 12 months of intervention and longitudinal change (12 months—baseline)

	Individual intervention (n = 76)	Collective intervention (n = 77)	*p-*value
Body mass index z-score			
Baseline	3.21 (3.10 to 3.32)	3.13. (3.00 to 3.2)	0.125
12 months	2.58 (2.46 to 2.70)	2.72 (2.61 to 2.83)	0.155
*p-*value	< 0.0001	< 0.0001	
Absolute change	− 0.63 (-0.72 to -0.54)	− 0.41 (− 0.51 to − 0.31)	< 0.001

Values are mean (95% CI)

At multiple stepwise analysis, only change in BMI z-score remained independently associated with intervention (individual *vs*. collective intervention, odds ratio 3.37, 95% CI 1.29 to 8.77, *p* < 0.001), that means a 5% (95% CI 2 to 8%) higher probability of a 1% BMI z-score reduction in individual *vs*. collective intervention. Statistical significance of change in AIP and TyG was *p* = 0.146 and *p* = 0.069, respectively.

No adverse events were reported in any participant during the study period.

## Discussion

This intervention study aimed to assess if promoting a normo-caloric and balanced diet together with physical activity in children with obesity may affect differently on the Atherogenic Index of Plasma in those undergoing an individual- or collective-based intervention. The study included a consecutive series of children recruited in a centre for childhood obesity activated in 2009 and accredited as an EASO Paediatric Collaborating Centre for Obesity Management (https://easo.org/coms-2/). The study had adequate power and the retention rate was satisfactorily high after 12 months (> 90%).

Intervention was primarily based on well-established and standardized nutritional recommendations [22–24]. Furthermore, in the individual-based intervention behavioural changes techniques from CALO-RE taxonomy were applied [25]. Overall compliance with intervention was acceptable, with 90% of children who assumed at 12 months within the recommended ranges of energy intake.

Only few studies evaluated the AIP in the general paediatric population [34, 35] and to our knowledge no trial was conducted in children with obesity. Nogay et al. [34] estimated in the subgroup of children with BMI ≥ 95th percentile a mean AIP of 0.01 and 0.05 in those aged 6–11 and 12–18 years, respectively. Higher mean values were found in this trial. Indeed, a direct comparison with results by Nogay et al. [34] could be misleading, as in this trial children exhibited at baseline a mean BMI z-score higher than 3, corresponding to a value over the 99th percentile [36]. Further, it is unclear if in Nogay et al. [34] the 95th percentile was referenced to the general population or estimated in the examined sample. Anyway, the findings would suggest a possible better effectiveness on the AIP of the individual intervention (mean reduction of 41.4% after 12 months) over the collective intervention (mean reduction of 18.5%), although the comparative size of the effect could be overestimated in this trial, as at baseline children in the individual intervention group showed higher total cholesterol and LDL cholesterol values than children in the collective intervention group. The potential clinical meaning of these findings would be possibly interesting, as the AIP reflects the relationship between protective and atherogenic lipoprotein and is associated with the size of pre- and anti-atherogenic lipoprotein particles [9, 11]. Dobiàsova and Frohlich [10] pooled together 35 cohorts for a total of 1433 subjects (children and adults) and found an association between AIP and cholesterol esterification rate in apoB-lipoprotein-depleted plasma (FER_HDL_), an indicator inversely related to the size of HDL particles [10]. In addition, these authors found a maximum AIP value of 0.12 in adults without high risk of coronary artery disease while the weighted pooled mean of AIP in subjects with high risk of or angiographically proven coronary artery disease was 0.27 and 0.30, respectively [10]. In children aged 5–14 years, all without metabolic diseases and obesity, the authors estimated a mean of AIP ranging from -0.09 (girls 5–9 years) to 0.02 (girls 10–14 years) [10]. In this study, the relatively high overall mean of AIP observed at baseline (0.28) supports that attention should be paid in the management of obesity in the paediatric population, and that interventions should be hopefully long lasting targeted and strictly monitored.

A decrease in triglyceride-glucose index was observed with better longitudinal change in the individual. Reduction of insulin, HOMA-IR and HOMA-β%, and increase of QUICK index occurred in the individual intervention group only. Overall, the results suggest a possible supplementary beneficial effect of the individual intervention over the collective intervention on TyG and insulin, suggesting an improvement of insulin sensitivity [35]. Other studies investigated TyG in children and adolescents [37–39], although they were not designed to evaluate the role of nutritional interventions in the obese paediatric population. Mohd et al. [38] reported in 225 obese adolescents with normal glucose tolerance, prediabetes, and type 2 diabetes, an association of the TyG index with in vivo insulin sensitivity, evaluated by the hyperinsulinemic–euglycemic clamp. Rodrıguez-Moran et al. [39] noted that the TyG index was highly sensitive compared to the euglycemic-hyperinsulinemic clamp and that it had a high diagnostic concordance with the HOMA-IR in a cohort of 2779 Mexican schoolchildren, as also observed by Kang et al. [37]. Moreover, it should be also considered that while HOMA-IR may mainly reflect hepatic insulin resistance, the TyG index may mainly reflect muscle insulin resistance [40]. Also, an increase in plasma triglycerides may interfere with normal glucose metabolism of the muscle, thus reducing insulin sensitivity [41].

Lastly, BMI z-score decreased in about 95% of children with a mean decrease of 0.63 (19.6%) and 0.41 (13.1%) in individual- and collective-based intervention, respectively. These findings agree with previous studies [35, 42–45], reporting after 5–12 months of nutritional only or lifestyle interventions in overweight/obese children a mean decrease of BMI z-score ranging from about 5% [43, 44] to 20% [43]**.** Interestingly, it should be noted that the effectiveness of an intervention on the cardio-vascular risk has been recognized when the BMI z score reduction is at least 0.5 [46, 47].

This study has some limitations that need to be highlighted. The main limitation arises from the unfortunate circumstance that unexpectedly, at baseline, children in the individual intervention group had significantly higher total cholesterol and LDL cholesterol values than children in the intervention group. This makes the quality of the trial sub-optimal, as the difference in these basic parameters of atherogenesis warns that drawing conclusions about the effect size of the individual intervention, could be partially misleading when not discussed carefully. On October 2020, we conducted a review of all the trial procedures. It was noted that the family paediatricians involved in the recruitment possibly did not homogeneously referred the children for the hospital visit. When they were re-contacted anonymously some of them admitted sometimes not to refer the child to hospital, as they deemed unnecessary at that time, contravening the criteria and recommendations they had received during a classroom lesson held one month before the start of the trial. This unpredictable and deplorable attitude may have introduced a hidden bias into the randomization procedure. Anyhow, this limitation deserves to be shortly discussed. Confidently, one could firstly remark that the AIP index is defined by TG and HDL and in turn the observed beneficial effect on this index in individual *vs.* collective intervention, might be, at least in part, overestimated in this trial, possibly due to baseline worse cholesterol profile exhibited by children in individual intervention group. Accordingly, it could be not unexpected that children in the individual intervention showed more beneficial effects. However, one should also note that in this study baseline AIP index (the primary outcome measure we used) did not differ at baseline between the two intervention groups (mean 0.29 in individual vs. 0.27 in collective intervention). Moreover, even hypothetically assuming the same mean baseline value of 0.27 the mean reduction in the individual group would be 0.10 (observed value 0.12) *vs*. the value of 0.05 observed in the collective group, and the difference would still be significant. This would suggest that the greater improvement in the AIP index in the individual intervention group may not only resulted from a *worse* baseline blood lipid profile, but, at least in part, from the type of intervention itself. Another question, related to the baseline differences in cholesterols, may arise concerning at the baseline carbohydrate intake of about 30 g higher in the individual intervention group, and if it may be a possible confounder. However, one should note that although studies suggest that while higher total carbohydrate intake, as percentage of calories from carbohydrate, can relate to blood cholesterol profile (*e.g*., [48–50]), also when %carbohydrate ranges around 50%, orientation of the association (negative/positive) is not currently conclusively clear. For example, Ma et al. [51] found at a cross-sectional analysis that percentage of calories from carbohydrates, as well as total intake of carbohydrates (on units of 20 g), were inversely associated with total cholesterol, LDL cholesterol and HDL cholesterol at unadjusted analysis. After adjusting for confounders (including BMI, percentage fats intake, season of year at lipids assessment and others) none of these associations remained significant (except HDL cholesterol/%carbohydrates, *p* = 0.03). On longitudinal adjusted analysis, they found a significant decrease in HDL cholesterol and increase in total cholesterol/ HDL cholesterol ratio related to %carbohydrate. On a whole, they concluded that "further studies in the form of clinical trials are required to investigate these associations and to disentangle the relationship between dietary carbohydrate intake and serum lipids". While these results were reported in adult population Nicklas et al. [52], assessing schoolchildren aged 7–11 years (a range of age comparable to children in our trial), observed that the effect of diet on serum lipids in children is similar to the one observed in adults. Not only, they reported also that total fat and saturated fat were positively associated with total cholesterol and HDL cholesterol levels, saturated fat was positively associated with total cholesterol, while carbohydrate was inversely associated with both total cholesterol and HDL cholesterol. Although it cannot be proved that the above considerations may be translated to children with obesity, in absence of specific trials they strongly warn that anyway a complex interrelationship might exist of carbohydrate intake with serum lipids, that needs to be purposely investigated in childhood obesity. Lastly, another limitation was that this study based the dietary assessment on the Food Frequency Questionnaire technique. However, one should note that it was used merely as a tool to estimate compliance with nutritional recommendations. Future research should hopefully include more accurate procedures, such as the 7-day food diary.

## Conclusion

On the whole, within the limitations of this study, one may conclude that in children with obesity, an individual-based nutritional and lifestyle intervention, including techniques from the CALO-RE taxonomy, could have an additional beneficial effect over a collective-based intervention, although the actual size of the effect remains to be clarified.

## Data Availability

The datasets generated and/or analysed during the current study are not publicly available due the raw information gathered in this research were kept strictly confidential as stated in respondents’ consent agreement but are available from the corresponding author on reasonable request.

## Abbreviations

AIP: Atherogenic Index of Plasma
WHO: World Health Organisation
CALOR-RE taxonomy: Coventry, Aberdeen and London- Refined taxonomy
BMI: Body Mass Index
MVPA: Moderate to vigorous daily physical activity
FFQ: Food Frequency Questionnaire
HDL: High-density lipoprotein
LDL: Low- density lipoprotein
TG: Triglycerides
ApoA1: Apolipoprotein A1
ApoB: Apolipoprotein B
HOMA-IR: Homeostatic models assessment of insulin resistance
HOMA-β%: Homeostatic model assessment of β-cell function
QUICK: Quantitative sensitivity check
TyG: Triglyceride-glucose Index
FER_HDL_: Fractional esterification rate of cholesterol in high density lipoprotein
EASO: European Association of the Study of Obesity
